# Pelvic orthosis effects on posterior pelvis kinematics An *in-vitro* biomechanical study

**DOI:** 10.1038/s41598-018-34387-7

**Published:** 2018-10-29

**Authors:** Stefan Klima, Ronny Grunert, Benjamin Ondruschka, Mario Scholze, Thomas Seidel, Michael Werner, Niels Hammer

**Affiliations:** 1 Orthopaedicus Clinics, Leipzig, Germany; 2 Department of Orthopedic and Trauma Surgery, University Clinics of Leipzig, Leipzig, Germany; 3 Fraunhofer Institute for Machine Tools and Forming Technology IWU, Dresden, Germany; 4 Institute of Legal Medicine, Medical Faculty University of Leipzig, Leipzig, Germany; 5 Department of Anatomy, University of Otago, Dunedin, New Zealand; 6 Institute of Materials Science and Engineering, Chemnitz University of Technology, Chemnitz, Germany; 7 Institute of Cellular and Molecular Physiology, Friedrich-Alexander University Erlangen-Nürnberg (FAU), Erlangen, Germany

## Abstract

The sacroiliac joint (SIJ) is a well-known source of low back pain, with increasing interest for both conservative and surgical treatment. Alterations in pelvis kinematics are hypothesized as a contributor to SIJ pain and pelvic orthoses one treatment option, but their effects on the pelvis are poorly understood. Alterations in movement patterns induced by the application of pelvic orthoses were determined in five human cadaveric pelvises. Deformations were obtained from the lumbosacral transition and the bilateral SIJ, using digital image correlation and a customized routine to compute the movements within the pelvis. Significant alterations were found for the movements at the SIJ, in particular a vast increase in axial (x-axis) rotation, accompanied by increased inferior (y-) translation of the sacrum relative to the ilium. Movement patterns at the lumbosacral transition changed, causing increases in axial rotation and decreased inferior translation of L5 relative to S1. Using a physiologic mode of load application gives novel insights into the potential effects of pelvic orthoses. The results of these *in-vitro* experiments vary markedly from previous experiments with loading limited to two or less axes. Furthermore, the influence of pelvic orthoses on the lumbosacral transition warrants further investigation.

## Introduction

The sacroiliac joint (SIJ) has been identified as an important source of pelvic girdle and low back pain^1–4^. Alterations in pelvic ring kinematics are partially made responsible for this pain entity, with particular focus on the ligaments of the posterior pelvis^5–7^. Previous studies have pointed out the role of ligaments in maintaining SIJ kinematics in close limits^4,8,9^, being a link between both form and force closure^2,10,11^. There appears to be a correlation between impaired pelvic ligament function and the onset of SIJ pain^11–14^. However, sparse scientific evidence exists proving this theory.

There is recent experimental data that impaired ligament function may alter pelvic ring kinematics, both mechanically^15^ and potentially also clinically^16^. Mechanoreceptors and free nerve endings have been described in the ligaments and adjacent cartilage of the posterior pelvis^17–21^. These fibers could be involved in neuromuscular feedback loops^22–24^ and be responsible for sustained pain in the posterior pelvis as a consequence of mechanically-induced irritation.

An established non-surgical approach to treat the painful SIJ is to apply pelvic orthoses^14,25–29^. A number of experimental studies have postulated that orthoses may relieve pelvic ligaments^7,30,31^ or decrease laxity of the SIJ, defined as an unphysiologic excessive movement within the joint^7,14,23,26^. These findings have been correlated to clinical findings on beneficial outcomes of orthosis applications^27–29^. However, to date, no study has shown how specifically SIJ kinematics become altered under orthosis application, and if the changes induced by orthoses are different in the healthy condition compared to the condition with insufficient ligaments.

The given study aimed at investigating the effects of pelvic orthosis on lumbopelvic kinematics using a human cadaveric model. Changes induced by orthoses were examined in the intact osteoligamentous lumbopelvises with physiologic load in a double-leg stance scenario.

It was found that the application of pelvic orthoses results in increased movements at the SIJ and altered kinematics of the lumbosacral transition as a consequence.

## Methods

### Tissues and mechanical setup

Six cadaveric pelvises were retrieved from human body donors after their passing. While alive, the body donors gave written and informed consent to the donation of their tissues for research and teaching purposes. All tissues were obtained in accordance to the the Saxonian Death and Funeral Act (version 2014). The mean age was 81.3 ± 10.0 years (3 males, 3 females). None of the tissues showed disease with impact on the musculoskeletal system and all were used in a fresh condition to exclude mechanical alterations on the bones and ligaments^32,33^. The pelvises were grossly removed from soft tissues, leaving the L5-sacrum transition and the pelvic ring intact with all adjacent ligaments as shown before^34,35^. For physiologic load application onto the pelvis mimicking double-leg stance, the L5 endplate and both acetabula served as contact points (DYNA-MESS, Aachen, Germany). An area measuring 1 cm^2^ was removed from the endplate of L5 before a metal pin was inserted into the vertebral body and cemented in with ceramic powder-reinforced polyurethane (RenShape solutions, Huntsman International LLC, Salt Lake City, USA). An indentation plate was mounted onto the metal pin. This construction was further connected with a spherical stamp component to the mechanical testing device. Adjustable AO titanium plates were further implanted to the iliac crest on both sides with AO standard screws of varying length. Steel wires attached to the titanium plates at the iliac crest were used to simulate muscle traction of the erector spinae and of the abdominal wall muscles. Match-sized femoral heads were used for both acetabula. Following 20 cycles of preconditioning ranging between 0 N and 20% of each donor’s body weight, 12 loading cycles between 0 N and 100% body weight were conducted at 150 N/sec. The experiments were conducted without pelvic orthosis and repeated with a size-matched commercially available orthosis mounted to the pelvis at a tension of 50 N^7,30^.

**Figure 1:**
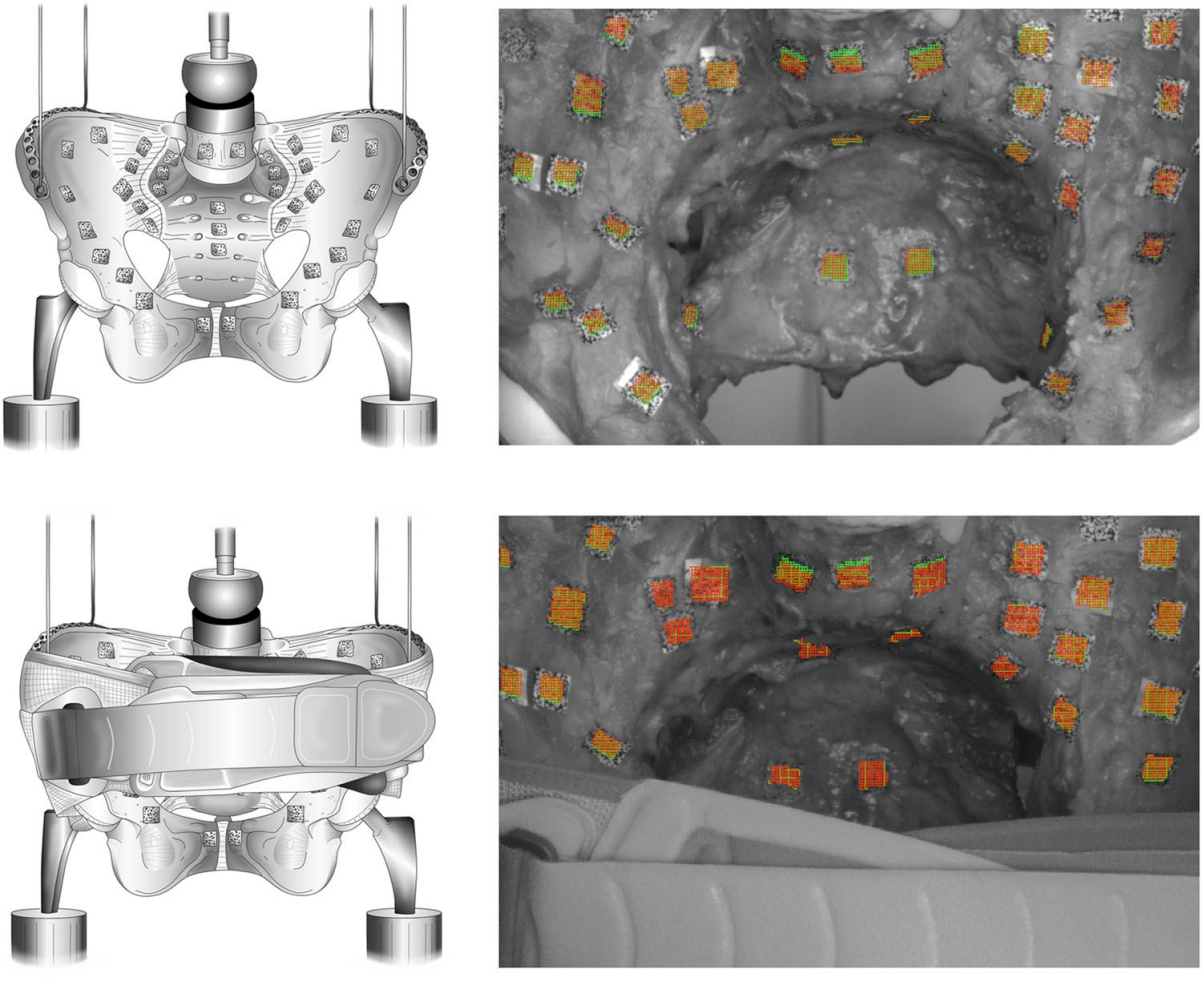
Summary of the experimental setup without pelvic orthosis attached (left top) and with applied pelvic orthoses. The right set of images shows the corresponding deformation data obtained with no orthoses (top right) and with an orthosis attached (bottom right). Peak deformations were observed at the lumbosacral transition and the sacroiliac joint, indicated by the deformation fields in the speckle markers from image correlation.

A Limess digital optical image correlation (DIC) system was utilized (Krefeld, Germany; precision 0.01 pixel or 1 µm, 5 fps, FOV 2.0 megapixels). Speckle markers were attached to the L5 vertebra, the sacrum and the ilium bilaterally. The image correlation was synchronized with the crosshead displacements of the testing machine. Rotations and translations were computed, aligning the optical data in local coordinate systems (Istra4D, Dantec, Skovlunde, Denmark), and a custom-programmed approximate approach routine (MATLAB, Mathworks, Natick, MA, USA). Data were evaluated for 20%-increments from 0 to 100% body weight of rotations and translations in x-, y- and z-axes. Positive values were defined as lateral in the x-axis, cranial in the y-axis and anterior in the z-axis. Movement thresholds for rotation and translation were defined as ≥ 0.1° or ≥ 0.1 mm, respectively. Fig. 1 summarizes the experimental setup.

### Data evaluation

MATLAB (v2017a, MathWorks, Inc., Natick, MA, USA) was applied to import the coordinates and displacements of marked triangle points (one triangle per region respectively three points per region – sacrum/L5/ilium) in both conditions, with and without orthosis. Furthermore, these digital triangles were set at exactly the same positions in both conditions, with and without orthosis, to maintain comparability. The corresponding displacements of the points were corrected for rigid body movement (rigid body motion removed, RBMR) at each time step directly in the DIC-software prior to export. The displacement-data sets were first noise-filtered by a moving mean filter with a centered window spanning 25 steps and for each triangle, the center of mass (centroid, *C*) was calculated. The relative displacement *s*_*rel*_ of a triangle *A* with respect to a second triangle *B* was defined as the difference in centroid displacements:

$${s}_{rel}={\rm{\Delta }}{C}_{A}-{\rm{\Delta }}{C}_{B}=({x}_{C,A}-{x}_{C,B},{y}_{C,A}-{y}_{C,B},{z}_{C,A}-{z}_{C,B}).$$

**Figure 2:**
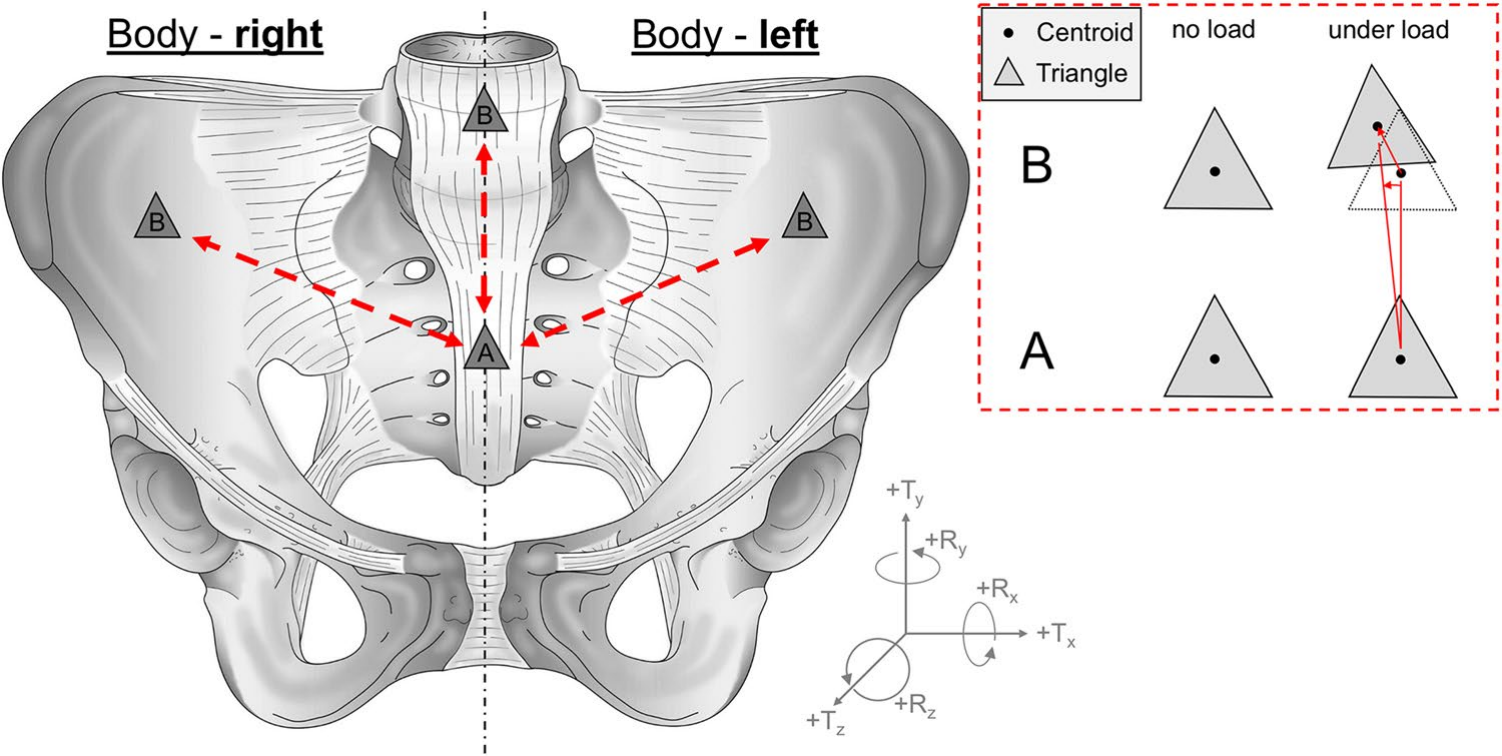
Overview of the regions used for the evaluation and a schematic movement of triangles under load. A displacement of the centroids as well as a rotation of the triangles were calculated from defined marker points. SIJ motions from the right body site were mirrored to the contralateral site.

An overview of the regions used for the evaluation and a schematic movement of triangles is summarized in Fig. 2. The displacements were used to compute both load- and body-weight dependent displacements as RBMR functions for the SIJ and the lumbosacral transition. Additionally, for each pair of triangles *A* and *B*, rotation of *B* around the centroid of *A* was calculated. The center of the Cartesian coordinate system was identified with the centroid of *A*. The points of *B* were registered for each time step relative to step 0 using the Iterative Closest Point Method algorithm implemented in MATLAB (pcregrigid). The transformation matrix was decomposed to obtain rotations around the *x*-, *y*- and *z*-axes. Both translations and rotations were solved from the rotation matrix in the order Z-Y-X. Body-right sited SIJ motions were mirrored to a body-left coordinate system under the assumption of a symmetric pelvis (translation in X direction and rotations around Y and Z multiplied by -1). The deformation curves were then scanned and evaluated via a second MATLAB routine. Movements were retrieved from the 12 load cycles as relative changes at a preload (50 N) = 0%, 20%, 40%, 60%, 80% and 100% of the cadavers’ body weight and as absolute changes at a preload (50 N), 100 to 500 N in 100-N steps. Data were further corrected for offsetting to each of the previous preloaded step. Means and standard deviations were calculated from the sampled points for every single region.

### Statistical evaluation

Data analysis was conducted using Microsoft Excel 2016 (Redmond, WA, USA), Prism 7 (Graphpad Software Inc., La Jolla, CA, USA) SPSS 23.0 (IBM, IL, USA). The D’Agostino-Pearson omnibus normality test was used to assess normal distribution, followed by the Friedman test and Dunn’s test for multiple comparison with post-hoc adjustment. *P-values* of 0.05 or less were considered as statistically significant.

## Results

Five pelvises gave results on the load-deformation behavior in the DIC in both conditions, i.e. without the orthosis and with the orthosis attached. From one pelvis (male 75-year-old), accurate load-deformation data was obtained only in the intact condition without the orthosis. With the orthosis attached, speckle markers were covered by the pelvic orthosis in this specimen, which required exclusion of the corresponding data set. The failure loads of the pelvises averaged 2145 ± 389 N (range 1941 to 2700 N), corresponding to 281 ± 13% body weight (range 249 to 346%), respectively. Sacral fractures were the primary cause of material failure in four pelvises and an L5 vertebral body fracture was the cause of failure in the fifth pelvis. Movements and movement alterations were observed in the sub-degree and sub-millimeter range and with a markedly high inter-individual variation, but equally high consistency of the movement cycles within each of the cadaveric pelvises. Measurement precision for the SIJ was 0.04 mm and 0.13°, 0.08 mm and 0.22°, and 0.04° and 0.09° in the x, y and z direction, respectively. For the lumbosacral transition, measurement precision was 0.01 mm and 0.41°, 0.31 mm and 0.11°, and 0.12 mm and 0.08° in the x, y and z direction, respectively.

**Figure 3:**
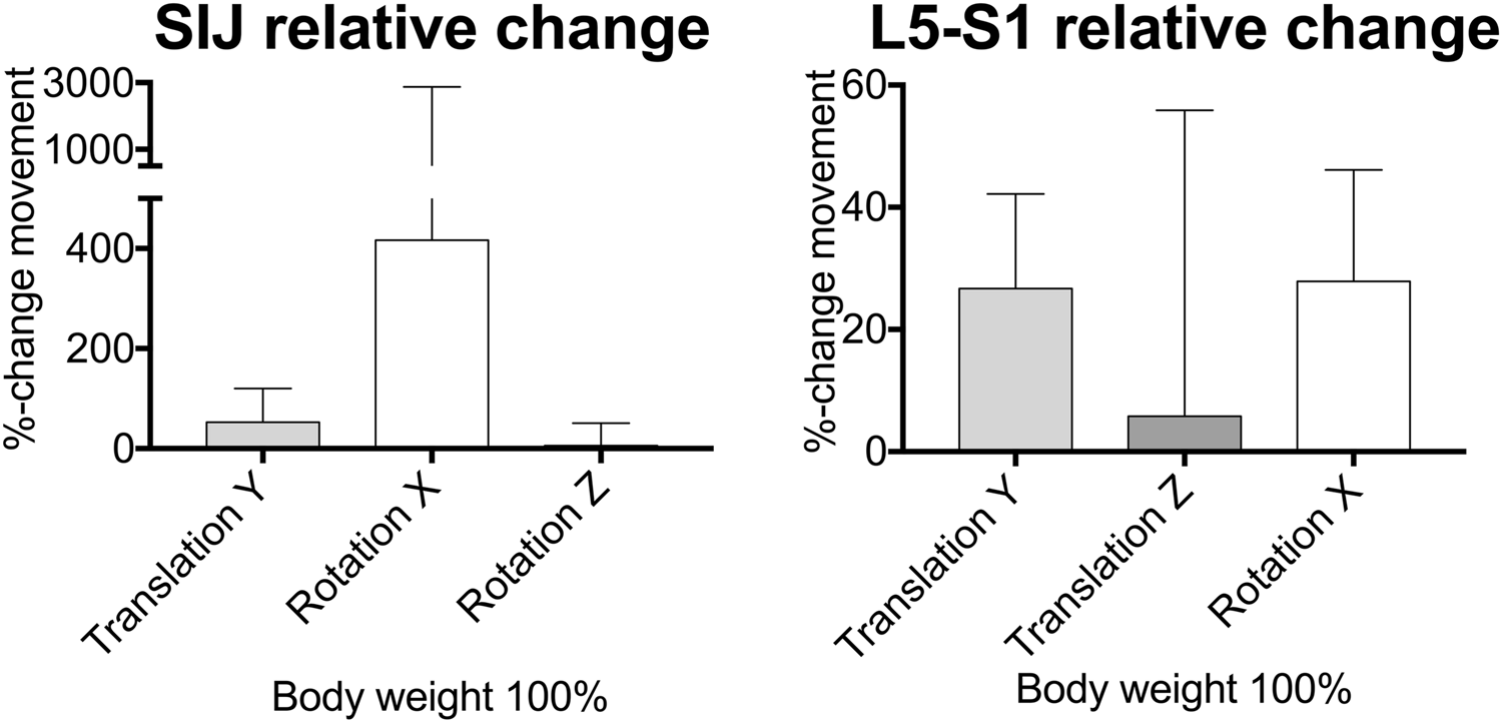
Bar graph summarizing the averaged data (absolute values) from changes induced by pelvic belt application in each of the pelvises under 100% body weight loading for the sacroiliac joint (SIJ) and the lumbosacral transition (L5-S1). The bars indicate the mean, the whiskers standard deviations. The quality and direction of the change in motion is seen in Figs. 4 and 5.

**Figure 4:**
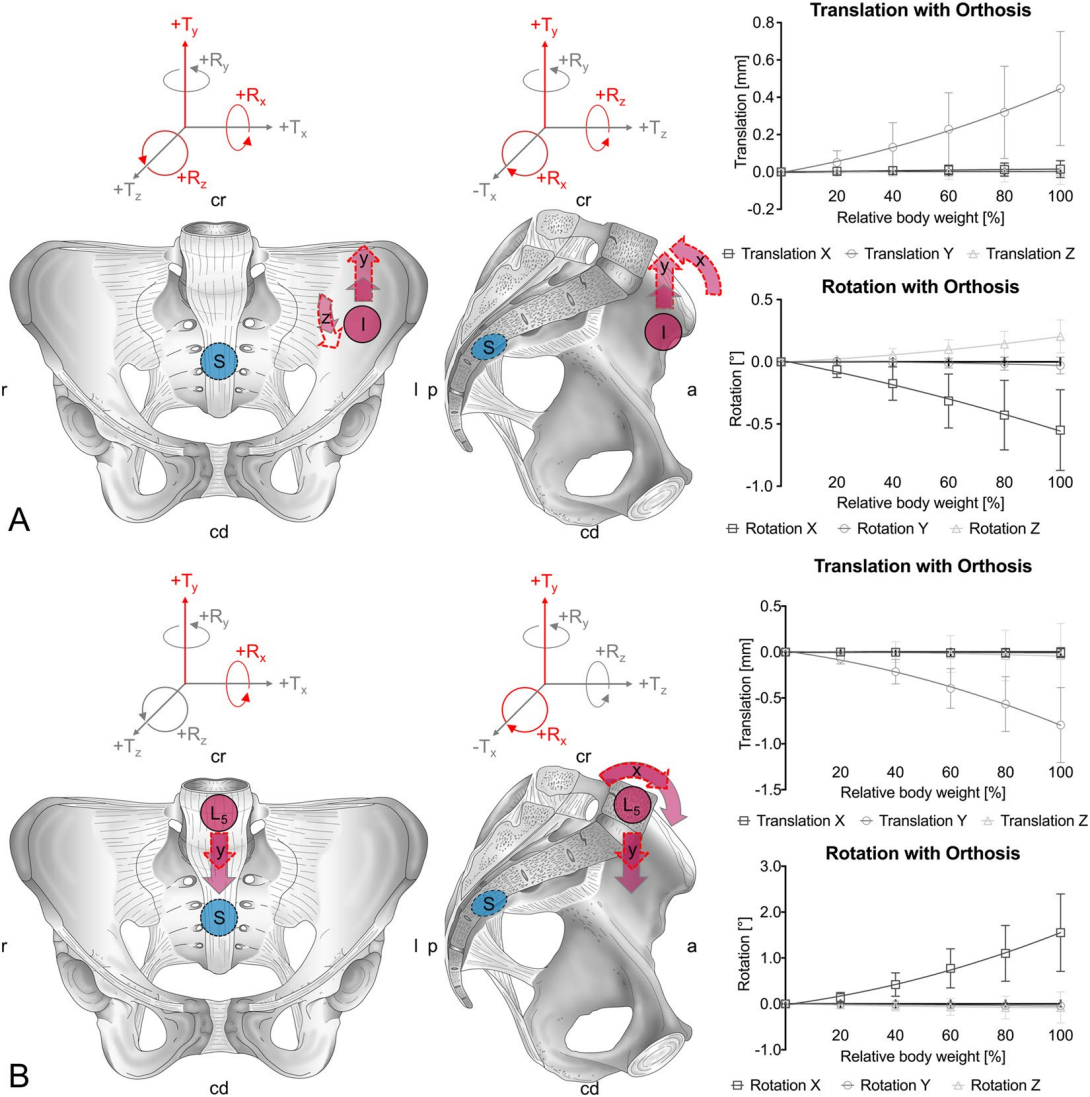
Graphical summary of the changes induced by pelvic orthoses under 100% body weight loading. The grey arrows indicate the initial movement, the interrupted red arrows indicate the extent of motion. The right side summarizes the loading-dependent deformation for 20% increments in body weight loading. (**A**) sacroiliac joint, (**B**) lumbosacral transition. I = ilium, L5 = fifth lumbar vertebra, S = sacrum; cd = caudal, cr = cranial, l = left, r = right; R = rotation, T = translation.

**Figure 5:**
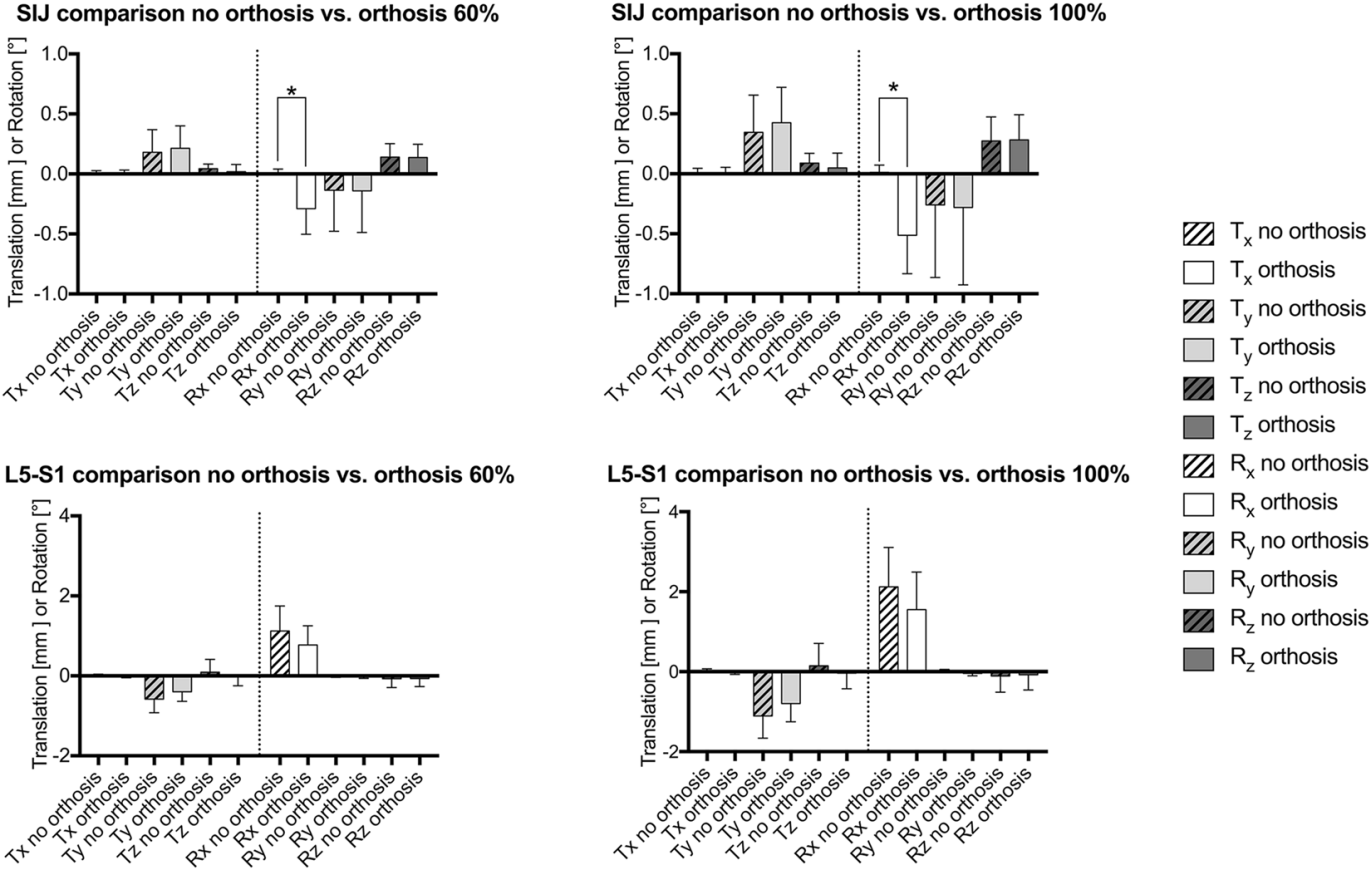
Bar charts summarizing the mean translation and rotation data prior to and following pelvic orthosis application under 60% and 100% body weight loading for the sacroiliac joint (SIJ) and the lumbosacral transition (L5-S1). The boxes indicate the mean, the whiskers standard deviations. Significant differences were observed for the axial (x-axis) rotation of the SIJ in spite of the large inter-individual variations in movements. *p values significantly different for R_x_ (60%) = 0.013, R_x_ (100%) = 0.038.

A non-linear behavior was observed for all motions, with an increase in the amplitude of motion at increasing loading. Relative changes of the individual movements are summarized in Fig. 3. Figure 4 gives an overview of the translations and rotations at 100% body weight loading and summarizes these movement alterations graphically. Figure 5 gives the mean values and standard deviations for all movements at 60% and 100% of the individual cadavers’ body weight. Supplement Figs 1–3 present the corresponding data under absolute loads ranging in 100-N steps until 500 N. Post-measurement precision was as follows at 500 N: T_x_ = 0.04 mm, T_y_ = 0.07 mm, T_z_ = 0.04 mm, R_x_ = 0.13°, R_y_ = 0.22°, R_z_ = 0.09° for the SIJ, and T_x_ = 0.01 mm, T_y_ = 0.31 mm, T_z_ = 0.12 mm, R_x_ = 0.41°, R_y_ = 0.11°, R_z_ = 0.08° for the lumbosacral transition. These values were used as reference for evident movement.

### Pelvic orthosis induce alterations in the movement patterns in particular in the axial rotation of the SIJ

Comparison of the individual changes in the pelvises’ movement at the SIJ yielded the following results for 100% of body weight: The inferior (y-) translation of the sacrum increased by 52%, axial (x-) rotation increased vastly by 416% in the sense of a counter-nutation (extension movement of sacrum), and (z-) rotation increased by 6% in the condition with an orthosis compared to no orthosis, respectively (Fig. 3). In spite of the high inter-individual variation in the movement patterns and the given sample size, the change in axial rotation was statistically significant at 100% body weight (p = 0.038), causing the changes in the minute rotations to become evident. Similar observations were made comparing the mean values averaged for all pelvises, showing inferior (y-) translations of the sacrum of 0.32 ± 0.31 vs. 0.45 ± 0.31 mm, axial (x-) rotations of 0.01 ± 0.05 vs. -0.55 ± 0.32°, and (z-) rotations of 0.16 ± 0.14 vs. 0.20 ± 0.13° in the conditions without and with orthosis, respectively (Figs 4 and 5).

### Corresponding changes to the SIJ were observed at the lumbosacral transition with decrease in axial rotation

At the same time at the lumbosacral transition, the following relative alterations in mean transition movements were observed: The inferior (y-) translation of L5 decreased by 26%, the anterior translation changed minutely by 6%, and axial rotation (flexion) decreased by 87% in the condition with an orthosis compared to no orthosis, respectively (Fig. 3). Similar observations were made comparing the mean values averaged for all pelvises, showing inferior (y-) translations of L5 of −0.97 ± 0.55 vs. −0.90 ± 0.41 mm and axial rotations of 1.82 ± 1.04 vs. 1.55 ± 0.84° in the conditions without and with orthosis, respectively (Figs 4 and 5). The anterior translation of 0.11 ± 0.47 vs. −0.04 ± 0.35 mm was evident but on the verge of measurement precision (threshold 0.12 mm)

## Discussion

### Pelvic orthosis application does alter SIJ and lumbosacral kinematics

This study aimed at quantifying the influence of pelvic orthoses on SIJ motion using a highly accurate *in-vitro* biomechanical model with physiologic loading. The model confirms published observations that SIJ motion is being altered by the application of pelvic orthoses and gives detailed insights in how this takes place. Moreover, it was observed that movement patterns at the lumbosacral transition are also altered as part of this complex chain of movement segments.

### Pelvic morphology appears to be largely decisive for SIJ kinematics, and there is evidence for innervated ligaments being potential target sites for treatment

It has been described that the SIJ does not have direct muscle attachments, but a range of muscles exerting compressional and torsional effects, including erector spinae, piriformis, gluteus maximus and the long head of biceps femoris^36,37^, acting onto the posterior pelvis predominantly via the posterior sacroiliac and the sacrotuberous ligament. Rotations and translations have been found to be the predominant movements occurring at the pelvis^8,38,39^. These movements are minute, and unlikely to be detected clinically^40^. The dense SIJ ligaments and the SIJ cartilage are limiting this movement. Changes in the SIJ cartilage, which would be indicative of degeneration or osteoarthritis in other joints, occur at a much earlier time point than in other joints both histologically^41,42^ and radiologically^42,43^. While it is well known that these changes in cartilage morphology are part of an adaption process to bipedalism, it is to date unclear if these changes are also related to the onset of SIJ pain. Differences in the osseous, cartilaginous and ligamentous morphology are likely to be related to different movement patterns, as could be observed in this given study.

Our findings underline multiaxial changes in pelvis movement including both the lumbosacral transition and the SIJ, with the effects of altered ligament loading. This change, however, appears not to be an exclusive limitation in the movements of the lumbopelvis, as especially the axial rotations increase at the SIJ in the context of a counter nutation movement (extension). One may hypothesize that the external loading to the pelvis causes decreased loading of the pretensioned SIJ ligaments and facilitates SIJ axial rotation and inferior translation of the sacrum relative to the ilium under loading. As a consequence, the inferior movement of L5 decreases, given that less of the inferior translation at the SIJ has to be compensated for, and potentially as a result of the load distribution via both the axial SIJ rotation and the complementary lumbosacral axial rotation.

### Do pelvic orthoses alter the ligament loading with potential effect on pain fibers?

Both ventral^44^ and dorsal^17,18,44–46^ spinal nerve rami innervate the SIJ, predominantly via the L4-S1 segments, thereby forming a fine network^44^. Further inputs are provided by the gluteal^47,48^, obturator^17^ and the pudendal nerve^48^. The cartilage^21^ and the ligaments^19,22^ are densely innervated, predominantly by mechanoreceptors^19–21^ and nociceptors^20,21^. Takasaki *et al*.^24^ and Jung *et al*.^23^ determined the effects of compression forces at the pelvis, ranging between 0 and 100 N on muscle firing patterns, and observed altered activity of the gluteus maximus and biceps femoris with increasing force. Different recruitment strategies appear to exist^49^. Kiapur *et al*. found that even small leg length changes may cause peak stress at the SIJ cartilage^50^.

Two potential scenarios could be the consequence for the innervation patterns and SIJ-related pain demonstrated by our results. The altered kinematics may either cause decreased pain receptor activation due to the less tensed ligaments, or the decreased movements at the lumbosacral transition may form part of the pain relief. This second scenario underlines the necessity to consider the SIJ not as an individual joint, but a component of a kinematic chain, which our experimental setup has considered.

### Load alterations induced by pelvic orthoses differ markedly from previous experimental and in-silico findings

There is published evidence that the force induced via pelvic orthoses decreases SIJ laxity and pain assumed to originate from altered lumbopelvic kinematics^24,26^. However, limited evidence exists that pelvic orthosis application reduced sacral mobility^25^. Previous biomechanical experiments have investigated the effects on the pelvis during both injury and surgical as well as non-surgical treatment, including cadaveric, virtual biomechanical and patient-control studies. Vleeming and coworkers^30^ investigated pelvic orthosis effects using a similar cadaveric setup as the given one with orthoses applied with a 50 N tensional load in bilateral stance. They found a significant decrease in axial rotation, accounting for 19%, which cannot be confirmed by our findings. Vleeming *et al*. furthermore hypothesized that ‘slackening’ of the dorsal ligaments may be a cause. The underlying conceptual model of ‘slackened ligaments’, however, is to date lacking in a morphological correlate, including altered ligament mechanics or changes in morphology related to SIJ pain. The experimental model used here was chosen according to previous observations on pelvic belt effects, regarding the mode of load application and tensional effects^14,30,51^. Our results on the effects of orthosis application show that SIJ translational and rotational movements increase. These experimental findings provide evidence that a slackening of the ligaments may not be exclusively causative, but rather overly stiff ligaments. This may potentially exert compression on the anterior joint regions, and consequently, may be relieved with increased axial motion.

Previous computational analyses have found that pelvic motion was largely influenced by ligament stiffness with site- and direction-dependent effects^52,53^. Here, beneficial effects of pelvic compression were determined, both by means of pelvic orthoses^7,31^ and surgical intervention^54–58^. While it is beyond the scope of the present study to focus on pelvic belt effects, it could be shown that our previous findings in finite elements simulation showing decreased SIJ axial motion and increased rotation around a sagittal axis may not be true in reality. Our results here furthermore indicate that our previous computer model may need further refinement to reflect the complex kinematics of the pelvis, and that movements in the relevant axes are likely to be at least one magnitude higher than assumed previously in the computer models^31^.

### Do pelvic orthoses have comparable effects like other intervention techniques? Experimental evidence

Injection techniques are commonly used to diagnose and treat the painful SIJ^59–65^, with fluoroscopy-guided techniques being more accurate^66^. Peripheral nerve stimulation^67,68^ and low-level laser therapy^69^ have evolved as treatment options. Similarly, nerve ablation techniques^70–75^ and surgical interventions using both open^76–78^ and minimally-invasive approaches^55–58,79^ have yielded promising results concerning patient outcomes^80^. However, comparative studies between the surgery and non-surgery groups found little advantage in long-term follow-up^81^, and surgery was accompanied by significant complication rates, especially infections^82,83^ and non-unions^84^. Here, pelvic orthoses appear to have certain advantages over invasive procedures.

Clinically, pelvic orthosis application appears to decrease pelvic laxity^23,26^. Moreover, beneficial effects of orthoses have been reported concerning decreases in pain perception^27^, health-related quality of life and postural steadiness^29^. Tension appears to have less effect than position^14^, and changes to pelvic morphometry overall appear to be minute^28^. The range of motion detected in our experiments confirms these findings, and sheds light on the lumbosacral transition as an integral part of posterior pelvis kinematics. Our results are consequently in line with previous experimental and clinical findings showing beneficial effects of pelvic orthoses to treat SIJ-related pain^29^. The results presented here, however, speak in favor of increased movement at the SIJ as a consequence of orthosis application, which is a novel finding that has previously only been postulated anecdotally. The clinical implications of this additional movement are largely hypothetical at this stage. They could be beneficial in regards to pain reduction, facilitating a more physiologic movement at the posterior pelvis, which is being impaired following (partial) failure of the SIJ ligament. Moreover, the effects on the lumbosacral transition should be considered carefully in light of potential lumbogenic causes of SIJ pain. An application of pelvic orthosis could alter the range of motion at the lumbar spine, to the effect that degenerative processes might be facilitated in this load-bearing region. This might even explain the anecdotal reports of increased pain following the application of pelvic orthoses. Such conclusions, however, remain largely hypothetical. In light of our experimental findings, the indication of pelvic orthoses should be set critically, following a thorough clinical examination under no influence of analgesics to rule out potentially negative effects. Future studies will need to investigate the clinical implications of the effects of pelvic orthoses on the lumbar spine in more detail.

### Limitations

The given experiments have a few limitations. Though the tissues were obtained in a supravital condition, removal of the soft tissues may have influenced lumbopelvic kinematics. The age of the body donors at death and the small sample size further limits the generalizability of the results, and our results warrant further quantification under conditions of impaired bone and ligament stability in a younger population, which is at higher risk of developing SIJ related pain. This study has not included the effects of pelvic belts on induced rotations to the lumbar spine and sacroiliac joint and this should be addressed in future.

## Electronic supplementary material

Supplementary figures
