# Supplementary material for: Pelvic orthosis effects on posterior pelvis kinematics An in-vitro biomechanical study
Source: Sci Rep. 2018 Oct 29;8:15980. doi: 10.1038/s41598-018-34387-7 (PMC6206162; doi:10.1038/s41598-018-34387-7)
Supplement: Supplementary file 1 — Supplementary figures [file 41598_2018_34387_MOESM1_ESM.pdf]

Pelvic orthosis effects on posterior pelvis kinematics

An in-vitro biomechanical study

Stefan Klima [1,2*], Ronny Grunert [2,3*], Benjamin Ondruschka [4], Mario Scholze [5], Thomas Seidel [6], Michael Werner [3], Niels Hammer [2,3,5]

1 Orthopaedicus Clinics, Leipzig, Germany

2 Department of Orthopedic and Trauma Surgery, University Clinics of Leipzig, Germany

3 Fraunhofer Institute for Machine Tools and Forming Technology IWU, Dresden, Germany

4 Institute of Legal Medicine, Medical Faculty University of Leipzig, Leipzig, Germany

5 Department of Anatomy, University of Otago, New Zealand

6 Institute of Materials Science and Engineering, Chemnitz University of Technology, Germany

7 Institute of Cellular and Molecular Physiology, Friedrich-Alexander University Erlangen-Nürnberg (FAU), Erlangen, Germany

* First and second author contributed equally to this publication

Corresponding author

Niels Hammer, M.D., Department of Anatomy, University of Otago, Lindo Ferguson Building, 270 Great King St, Dunedin 9016, New Zealand; Phone: +64 3 479 7362, Fax: +64 3 479 7254

Email: nlshammer@googlemail.com

Conflict of interest statement

The authors declare that no conflict of interest exists related to the given study, including honoraria, travel to conferences, consultancies, stock ownership (excluding publicly owned mutual funds), equity interests, and patent-licensing arrangements (particularly if a commercial product is noted in the article).

Supplement figures

Supplement figure 1: Box plots summarizing the averaged data from changes induced by pelvic belt application in each of the pelvises under 500-N loading for the sacroiliac joint (SIJ) and the lumbosacral transition (L5-S1). The boxes indicate the mean, the whiskers standard deviations. The quality and direction of the change in motion is depicted in supplement figure 3.

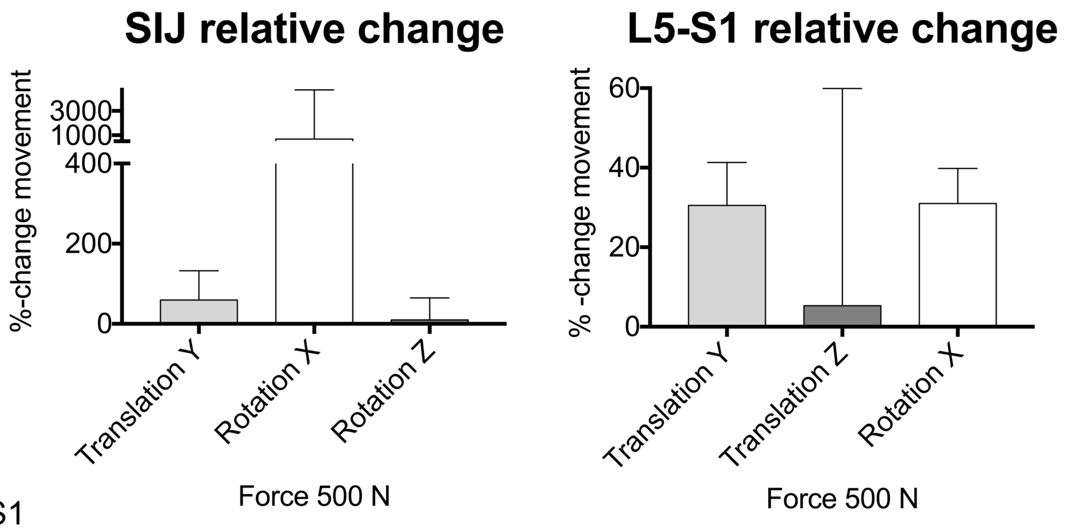

S1

Supplement figure 2: Graphical summary of the changes induced by pelvic orthoses under 500-N loading. The grey arrows indicate the initial movement, the interrupted red arrows indicate the extent of motion. The right side summarizes the loading-dependent deformation for 100-N increments in body weight loading.

A: sacroiliac joint, B: lumbosacral transition. I = ilium, L5 = fifth lumbar vertebra, S = sacrum; cd = caudal, cr = cranial, l = left, r = right; R = rotation, T = translation.

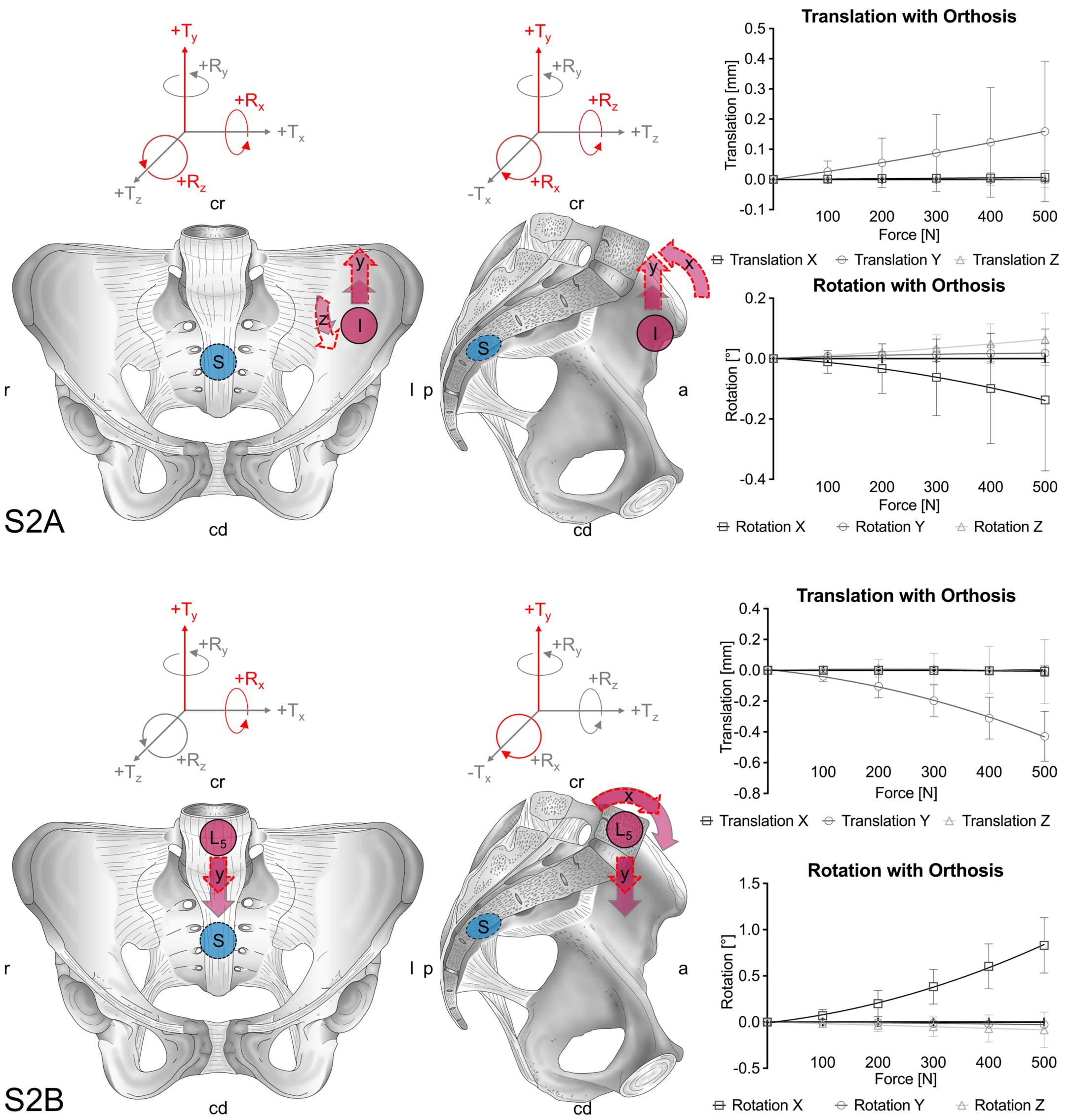

Supplement figure 3: Box plots summarizing the mean translation and rotation data prior to and following pelvic orthosis application under 300-N and 500-N loading for the sacroiliac joint (SIJ) and the lumbosacral transition (L5-S1). The boxes indicate the mean, the whiskers standard deviations. * *p value* significantly different for R_x (300 N) = 0.018, R_x (500 N) = 0.016.

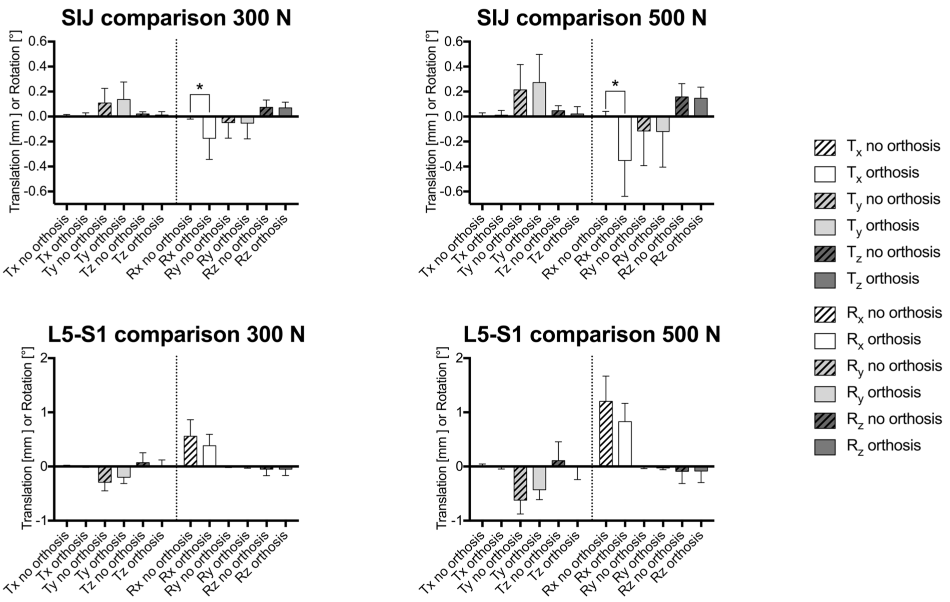

S3
